# Annotations

**Published:** 1897-05-29

**Authors:** 


### Annotations.

### Professional Badges.

A very old suggestion crops up again in a new form in a letter to a contemporary urging that medical men should have the privilege of wearing a badge on the right breast, the colour varying according to qualification, college, or university! It is pleasant to think that at last a medical man is found so proud of his profession as to wish to publish to all observers the fact that he belongs to it. Of course, the idea of the doctor carrying a label is capable of infinite expansion. If a man is not only to have a red lamp over his door to show his constant readiness to run when called for, but is to wear a badge by which he may be picked out of the crowd whenever a fancied emergency arises, it would no doubt also be found useful by the public that his wife should wear upon her bonnet some appropriate indication that she is ready to receive messages for the "doctor" as she goes her walks. His children also might be provided with little satchels, into which written orders might be popped as they pass on their road from school. The great problem of how to find a doctor would thus be solved at once! Moreover, when found, think of the great advantage of knowing by his button what sort of a man he was! We can only suggest as a final improvement that the badge should indicate the fee required and the length of credit given. How far some men are led by their love of decorations! Surely the author of this notable suggestion can see that all the decent doctors would put the wretched thing into their pockets so as to walk in peace, and that an astute public would soon extract the true meaning from the badge, and would read it as indicating want of work.

### Powder and Paint.

When we hear of fine ladies painting and powdering their complexions, and are told of the secret arts of the enameller, we are perhaps too apt to say that these attempts to imitate a beauty which does not exist are but proofs of the artificiality of modern life. No greater mistake could be made. The truly beautiful do not puff. It is the consciousness of a deficiency which leads to all these devices, and nothing can be more common among even the lowest intelligences, nothing can be more "natural," if one may use the term, than the tendency to hide peculiarities and deficiencies. Even among lunatics this sort of instinct shows itself. According to Dr. Claye Shaw, the striving to put oneself in accord with nature—in other words, to rectify deformities—is common in the insane, especially among the women. The hair ought to be glossy, therefore, in the absence of other means to produce this characteristic, they purloin the mutton fat, or pretend to be constipated so as to get some castor oil, by which to produce the desired effect; or when their complexions are not all that they would desire, they boil down their stockings so as to get the red dye out of the county mark, because that is their only substitute for rouge, and they elevate common whiting to the dignity of *poudre de riz*! And all because bruising and blotching must be hidden up! Perhaps even the savages who daub themselves with war paint do so to hide the bruises which warfare produces. Nay, it is even suggested that scarlet is chosen as the colour of our soldiers' uniform because the exciting effect of the flow of blood is not so clearly seen upon it as upon a background of other colours. The use of puffs and powders, and other facial decorations, is not then a matter of pure vanity, or æsthetic culture, but is a mere manifestation of the savage instinct which leads all creatures to hide every mark by which they differ from the normal.

### An Opportunity for the Apothecaries' Hall.

An audacious attempt is being made to induce the English press to step beyond even the very wide limits which modern journalism has set itself in dealing with quack advertisements. The scheme is this: An advertisement is written containing the most absolute and positive statements in regard to the wonderful curative powers of certain nostrums; then a newspaper is approached, and a big advertisement is arranged for, and then comes in the novelty, viz., the distribution of a large quantity of free samples of one of these nostrums, under the supervision of the newspaper in question, the arrangement being that the "results," as detailed by the silly people who take the stuff, shall be published for the edification of a gullible world. From the examples given of the sort of press notices which various American papers have been led by this system to give to these daring advertisers, we can see the lowering tendency of the whole performance. Meanwhile perhaps these good people will find that they have overshot their mark, for they not only advertise their various "cures," but advertise consulting-rooms as well, so that we may presume that someone is going to tell the trustful public which "cure" to take, in which case, if they sell the stuff at the same place, undoubtedly the rights of the Apothecaries' Society will be infringed. But what a prostitution of the press it seems when newspapers sell themselves—their paper, their type, their offices, and even their editorial staff—for the puffing of some quack nostrum. Is it that some mysterious affinity exists between medical, social, and political quackery, and that the general veracity of a paper is to be judged of by the character of its advertisements? Certainly the sort of readers who revel in bunkum on one side of a sheet seem generally to have a wondrous appetite for quackery on the other.

### The Workmen's Compensation Bill.

It has been sought to give power to the Committee on this Bill "to insert provisions to secure compensation to workmen for injuries to health arising out of and in the course of their employment." The Bill, however, expressly refers to injury from accident, and it has been decided to leave it so limited, the power asked for being refused. Only those who know how largely health is necessarily affected by certain trades will recognise how completely the inclusion of injuries to health would have altered the character and extended the operation of the Bill. It is unfortunately the fact that there are industries to enter which lead almost certainly to deteriorated health and shortened life; and equitable as it may be that a workman should be compensated for all injury to health caused by his occupation, such a provision would entirely alter the whole aspect and scope of this Bill. In the trades we allude to, injury to health, more's the pity, is no accident.

